# Effects of Guideline-Based Training on the Quality of Formal Ontologies: A Randomized Controlled Trial

**DOI:** 10.1371/journal.pone.0061425

**Published:** 2013-05-07

**Authors:** Martin Boeker, Ludger Jansen, Niels Grewe, Johannes Röhl, Daniel Schober, Djamila Seddig-Raufie, Stefan Schulz

**Affiliations:** 1 Institute of Medical Biometry and Medical Informatics, Albert-Ludwigs University Freiburg, Freiburg, Germany; 2 Institute of Philosophy, University of Rostock, Rostock, Germany; 3 Institute for Medical Informatics, Statistics and Documentation, Medical University of Graz, Graz, Austria; University of Catania, Italy

## Abstract

**Background:**

The importance of ontologies in the biomedical domain is generally recognized. However, their quality is often too poor for large-scale use in critical applications, at least partially due to insufficient training of ontology developers.

**Objective:**

To show the efficacy of guideline-based ontology development training on the performance of ontology developers. The hypothesis was that students who received training on top-level ontologies and design patterns perform better than those who only received training in the basic principles of formal ontology engineering.

**Methods:**

A curriculum was implemented based on a guideline for ontology design. A randomized controlled trial on the efficacy of this curriculum was performed with 24 students from bioinformatics and related fields. After joint training on the fundamentals of ontology development the students were randomly allocated to two groups. During the intervention, each group received training on different topics in ontology development. In the assessment phase, all students were asked to solve modeling problems on topics taught differentially in the intervention phase. Primary outcome was the similarity of the students’ ontology artefacts compared with gold standard ontologies developed by the authors before the experiment; secondary outcome was the intra-group similarity of group members’ ontologies.

**Results:**

The experiment showed no significant effect of the guideline-based training on the performance of ontology developers (a) the ontologies developed after specific training were only slightly but not significantly closer to the gold standard ontologies than the ontologies developed without prior specific training; (b) although significant differences for certain ontologies were detected, the intra-group similarity was not consistently influenced in one direction by the differential training.

**Conclusion:**

Methodologically limited, this study cannot be interpreted as a general failure of a guideline-based approach to ontology development. Further research is needed to increase insight into whether specific development guidelines and practices in ontology design are effective.

## Introduction

Formal ontologies are software artefacts that represent theories which attempt to give precise mathematical formulations of the properties and relations of certain entities [1]. In the biomedical domain, formal ontologies have been promoted as a key resource to enable knowledge management. Typical applications are semantic annotations of experimental data (e.g. high-throughput data from bioassays), information extraction applied to clinical documents, as well as the integration of heterogeneous databases and services [2]–[5]. A large number of biomedical ontologies have been created for a wide range of subject matters, and many of them have successfully been used, particularly in genetics and proteomics [6], [7]. In the meantime the support of formal ontology as an engineering discipline has been consolidated, and repositories for the standardized access to ontologies have been made available [8]–[11].

However, the use and re-use of ontologies is often limited due to insufficient representational and formal quality. A variety of errors in ontologies and their consequences have been described [12]–[18]. Principal causes for these problems are the complexity of ontology engineering together with the limited availability of trained ontology developers.

A skilled ontology developer should have practical knowledge in four key areas. Potentially, the most important knowledge source required for successful ontology engineering is the domain itself which is to be represented. Without a deep and sound understanding of the domain entities and the relations between them, ontology developers have nothing they can even start with. For instance, they will just fail when they have to face the task of building a correct taxonomic hierarchy of classes of things in an unknown domain, even before it comes to the design of more complex structures like partonomies, i.e. models that relate parts and wholes. Domain entities like objects, processes, qualities, functions etc., together with the relations that connect them can only be represented after acquiring a thorough understanding of the domain. Even though, there is a considerable risk to design formally correct ontologies which however miss the very point the ontology developer intended to be represented, thus ending up in a bad representation of reality.

The second requirement for successful ontology development is the mastery of the formalism used, i.e. the logical expression language, with detailed knowledge on how the language is composed (syntax) and what the expressions in this language mean (semantics). The formality and rigidity of logics is one of the largest obstacles for developers that come from their own domain, without having a mathematics or philosophy background. Experienced domain expert generally have no difficulty to express their knowledge in precise textual statements. However, human language allows for subtle distinction to express the whole range from universal truths to contingent facts. Formal ontologies, however, are restricted to formulate what is universally true in a domain. Many pieces of domain knowledge are outside the scope of formal ontology. To recognize this limitation requires training and experience. Furthermore, human language is misleading. Often, even basic syntactic constructs like head nouns with adjectival modifiers require diverging interpretations: a complicated pregnancy is a pregnancy, but a suspected pregnancy isn’t. Nevertheless we have seen that even experienced ontology developers are influenced by the idiosyncrasies of natural language and introduce inadequate meaning to formal constructs. Visual tools like the Protégé Ontology Editor allow to hide the bare formalism of logic-based description languages like the Web Ontology Language (OWL) from the developer. However, the ontology developer should be fluent in using the corresponding editor functions and know the meaning of the constructs of the formal language.

On top of the representational language, an ontology developer should be able to build on existing specifications and standards. A large body of consolidated frameworks on how domain entities should be categorized and represented is provided by various disciplines like philosophy, mathematics and information sciences, in particular top-level ontologies and ontology design patterns (ODPs). Top-level ontologies, like BFO, DOLCE or BioTop provide a foundation of foundational categories (e.g. process, material object, quality) and relations (e.g. part-of, participates-in), to be extended and specialized by domain ontologies. Ontology design patterns are inspired by so called software design patterns originating in software technology [19]–[20] forming complex elements of software code to be re-used in development scenarios with similar requirements or functionality. Both a top-level ontology and ontology design patterns provide re-usable building blocks to guide the development of new domain ontologies and to sustain ontology standardization and interoperability.

The development of an ontology is often driven by its later application. Here, the developer should understand the technical framework in which the ontology is to be embedded. This can range from the support of natural language processing to interoperability frameworks and visual navigation tools. For each application scenario, different features of an ontology might be important. While the segment of reality represented in an ontology remains constant across applications, the scope, the depth and the implementation of the representation is influenced by the use-case.

While the core knowledge necessary to develop ontologies originates in the domain to be represented, domain knowledge has to be connected with knowledge about the semantics of the representational languages and the ontological framework in which the representation is grounded, i.e. basic categories, relations, and constraints. Therefore domain experts who develop ontologies should stand on a solid ground in computer science, logics, and philosophy, either by teaming up with experts from these disciplines or by acquiring related skills and knowledge by targeted training.

Guided by this rationale, we have developed a guideline on good ontology design. It comprises what we consider to be the most essential knowledge to develop good quality ontologies (GoodOD guideline (http://purl.org/goodod/guideline)) using description logic (DL) for representation and reasoning. This guideline combines foundations from philosophical ontology and logics, with the representational language OWL DL, and is rooted in the domain top-level ontology BioTop [21]. The guideline itself is not intended as training material for beginners in the field of ontology engineering but as a concise report which can be used for consultation on the constituents of good ontology development. To actually provide domain experts with the knowledge from the GoodOD guideline, we implemented it in a curriculum [22], [23].

The aim of this study was to provide evidence that guideline-based training of ontology developers enhances their performance. Therefore, the effect of training of a certain problem solution based on our guideline was tested against the effect of training in ontology design that was not specific for this problem. This was done as a randomized controlled trial with students from the biomedical domain with a background in computer sciences. A set of ontology similarity (distance) metrics for ontologies was used as a measurement instrument. As primary outcome measure, similarity metrics were applied to compare the ontology artefacts created by students with a gold standard artefact created by experts. As a secondary outcome measure the intra-group ontology similarity was compared between groups. We assumed that an improved quality would manifest itself in two ways: (i) as a higher similarity of trained students’ artefacts to expert ontology artefacts, and (ii) in a larger similarity between products of trained students compared to products of untrained students.

### Quality of Ontologies in the Biomedical Domain

Ontologies use logic to formulate statements about what experts consensually assumed to hold true in a domain, with scope and granularity varying according to the use cases for which the ontologies are created.

To evaluate their quality is inherently difficult, as it depends on the definition of quality, as well as on the availability of measurement instruments (see below). The literature about quality problems in domain ontologies is rich, but difficult to be operationalized. Design errors described in the literature can be categorized into different types of various complexities:

- The violation of implicit or explicit naming conventions [24] result in lexical names of classes or relations which are likely to be misinterpreted or easily to be confused by humans.
- Other errors arise through the mix-up of commonsense meaning of natural language expressions with the semantics of the representational DL language. E.g., on the background of natural language pragmatics a value restriction like ‘*OxygenMolecule* **hasPart** only *OxygenAtom*’ can be wrongly understood to imply the existence of parts when seen throuth the lens of natural language pragmatics [18]. However, as a DL expression, a value restriction only limits the possible target classes of a relation without implying the existence of a relatum, i.e. something that is related. Hence, our sample statement says that the parts can only be oxygen atoms (which is wrong, as oxygen molecules also have protons as parts), without stating that every oxygen molecule consists of oxygen atoms (which is true but needs a different logical statement).
- Other failures are due to problems with the counterintuitive semantics of DL. We are used to understand that the absence of a statement on a fact does imply its negation. This is the commonsense interpretation throughout many applications of daily life and even in science. In description logic the absence of a statement from an ontology, i.e. an axiom, does not imply its negation (the so called *open world assumption*) with all its consequential implications.
- A very common error is the absence of explicit statements that things cannot be in the same class but are in disjoint classes: mutually disjoint partitions are not made explicit [3]. For instance, if an ontology developer creates the two sibling classes *Animal* and *Plant*, nothing is said about whether things can exist that are plants and animals at the same time. Although or just because of the small number of language elements and its consequential restricted expressivity, ontologies based on DL can contain a multitude of logical inconsistencies [25].
- A large and important class of errors is related to inadequate representations of the underlying reality itself that do not surface as inconsistencies or contradictions in a logical sense. They are mostly due to an incomplete or wrong understanding of the domain which is to be represented in the ontology. Consequently, they result in underspecification and inaccuracy regarding the underlying ontological premises. These errors are difficult to detect because the ontology reveals no logical contradictions when checked by a DL classifier. Only domain experts with a sound ontological background can detect these errors. They are able to understand the meaning of the critical representation as wrong underlying assumptions. In many cases, faulty ontologies were built without making the underlying assumptions explicit.However, when there are competing or incongruent ontological commitments in the background, they can be even more problematic [26], [27].

Not only the erroneous axioms themselves, but (potentially even worse) also their computed entailments have impact on basic tasks like equivalence detection, ontology alignment, and affect interoperability in general. Above all, ontologies are often not easily applicable in usage scenarios for which they have not been developed. An essential condition for the overall success of an ontology is that it can be used across a variety of use-cases and applications, and that it can seamlessly interact with other semantic resources.

Recently, some activities have emerged that target assurance and improvement of ontology quality. Coordination efforts from policy and best practice providers [8] assure the quality of ontologies provided by repositories [9], [10] and tools support the detection and correction of typical errors [17], [24].

### Training in Formal Ontology

A variety of educational material is available on ontology, logics and knowledge representation. It is presented in different forms like textbooks, tutorials, online courses, and lectures and has been written to facilitate ontology engineering for a broad range of learners from different domains. This material is mostly technically oriented and focuses on certain skills, e.g. the usage of Protégé editor or how to represent a specific domain for a specific usage scenario. A public repository especially of training material dedicated to OWL and bio-medical ontology is available at the website of the CO-ODE project (http://www.co-ode.org). Among introductory and expert material on the development of ontologies with the famous Protégé-OWL tutorial is linked using a Pizza-ontology as an example [28].

Sources for training material on ontology theory and ontology engineering are websites of individual researchers, most prominently the website of Barry Smith at the National Center for Ontology Research (NCOR, http://ontology.buffalo.edu/smith/). At the National Center for Biomedical Ontology (NCBO) some audio-visual material is available esp. on the usage of BioPortal [9].

It is not possible to mention and evaluate all available educational material on ontology and ontology development here. Most of the material is of high quality and is the result of long term educational efforts, which cannot be valued high enough. But many of these resources are focused on only one aspect of ontology, e.g. describing the expressivity of representational languages in the context of editing and reasoning tools, typically description logics with the Protégé editor and plugin reasoners. Other materials tend to be agnostic of the principles we formulated above like the use of existing upper-level ontologies and design patterns.

The curriculum we evaluated in this study integrated everything we considered to be necessary for successful ontology development beyond the knowledge of the domain itself. It is targeted especially to those we consider the most important future ontology developers, *viz.* students with a domain background in the life sciences. Furthermore, the curriculum is based upon an explicit guideline on good ontology development which we believe provides a necessary, consistent and sufficient core definition of good ontology design. All modules of the curriculum are built up on each other and use consecutive exercises and examples from the same domain ontology.

### Ontology Evaluation

There are numerous accounts of the evaluation of ontology artefacts. However, objectives, outcome measures and evaluation methods differ on a large scale, as they need to be aligned to the variant semantic flavours and expressivity, which in turn depend on the ultimately envisioned use case scenarios. Some quantitative metrics and frameworks for quality measurement of DL ontologies are available [29]–[31]. All of them are highly synthetic and take into account features of the ontology that – prima facie – seem to be correlated with its quality (such as connectedness, depth of the hierarchy, annotation coverage, etc.).

These metrics are often fraught with the problem that they are not validated independently, a fact that is largely due to the unavailability of a generally agreed upon and easy to operationalize account of ‘ontology quality’. Hence no simple objectified quality assessment method has emerged so far. As a consequence, inter-rater reliability between human experts assessing the ontologies is often the method of choice for determining ontology quality [18], though it is infeasible for larger quantities of ontology content.

As mentioned, the perceived quality characteristics of an ontology are very often dependent on the use case they were designed for. Some authors conclude that “[r]esearch results in biomedical ontology should always be evaluated against a biomedical task for which the ontologies are intended” [32]. This places ontology engineering in a light quite similar to software engineering, and puts the focus of ontology quality assurance on areas similar to quality assurance for software artefacts, which is also reflected in the adaptation of the SQuaRE quality model [31], [33]. We agree that usability is a key criterion for the successful adoption of ontologies in real world applications and firmly believe such approaches to be generally laudable since they try to address the needs of ontology users. Nonetheless, an exclusive focus on usability and use-case oriented quality criteria may fall short of capturing the fact that – to some extent – ontologies are more like standards than like software artefacts.

In some areas of ontology research, these problems are less pressing. For example, the quality of ontologies generated automatically by machine learning or text mining procedures or of automatically derived ontology alignments [34], [35] can be readily assessed by their similarity to a manually curated gold standard which is normally done, e.g., in competitions for text mining algorithms. To our knowledge there are no prior studies that apply this approach to the assessment of ontology artefacts produced by students after differential training against expert models in order to quantify the effects of the training.

## Methods

### Curricular Development and Implementation

The curriculum was designed following a six-step approach, which has been designed for medical education [22], [36] but is not limited to this domain. These steps are general needs assessment, needs assessment of targeted learners, goals and measurable objectives, educational strategies, implementation, and evaluation and feedback.

The definition of educational objectives is crucial for every educational process. Educational objectives essentially depend, among other factors, on the general requirements, the specific requirements of targeted learners and subject matter. Regarding the former two requirements, we outlined the general need to improve the ontology development quality and we have elucidated which components are necessary to achieve this goal. The needs of targeted learners who are domain experts and students from the life sciences depend heavily on their knowledge of computer sciences and formal logics, as well as on the subject matter they intend to represent. Since we did not want to overburden the curriculum, we restricted the group of targeted learners to students in life sciences with a background in computer sciences for which we could take basic knowledge and skills for granted.

The subject matter addressed in the learning objectives had been operationally defined by the GoodOD guideline which comprises the basics of philosophical and formal ontology, descriptions logics, minimal metadata recommendations, top level ontologies and ontology design patterns, intended as a basic toolkit for ontology developers in any domain. Furthermore, learning objectives were formulated addressing the prevention of ontology development errors described in the introductory section of this paper.

As an activity, ontology development requires the learners to acquire and shape knowledge of the domain to be represented, master the representation formalisms, as well as the skills of how to actually *perform the task* of creating a formal model. Therefore, we chose an instructional format which actively engaged the learners in a sequence of brief hands-on exercises, to be done individually or in small groups. Classroom lectures were minimized to short introductory talks. Every step of the curriculum was accompanied by printed handouts. Each instructional unit had duration of 105 minutes, each day consisting of four units. The complete seminar was designed for five and a half instruction days, corresponding to 22 units.

**Figure 1:**
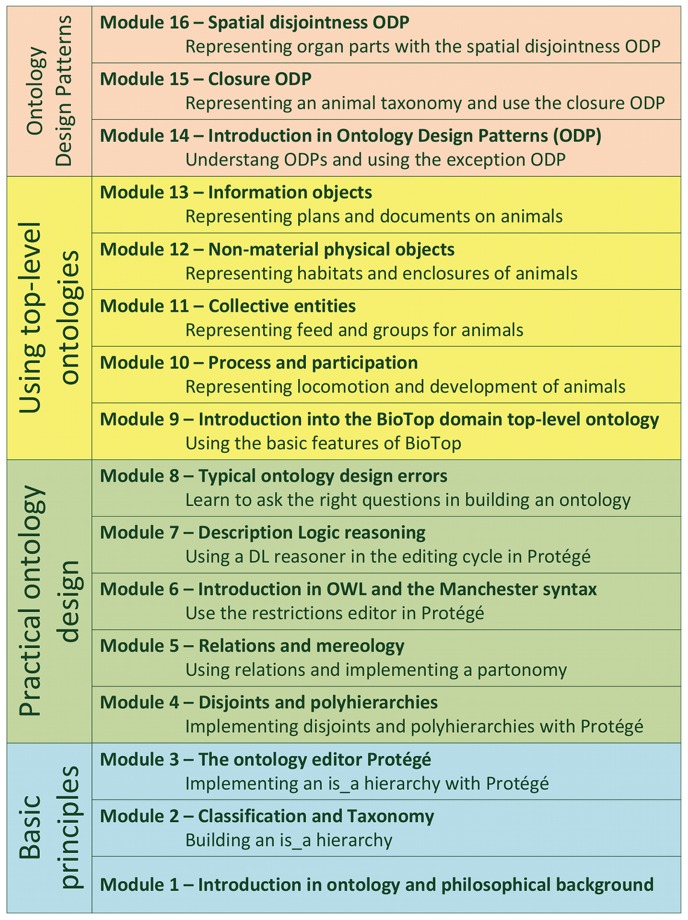
The sequence of modules in the curriculum. It follows the stepwise layout of the GoodOD guideline and the increasing complexity of the contents. Modules 10–13 and 15–16 were used in the intervention (see Table 1).

The material was subdivided into modules which loosely followed the structure of the guideline. Modules were assigned to one or two units of the instructional format described above. The topics of the individual modules were ordered by inter-module dependence, increasing complexity and difficulty. Figure 1 shows the module sequence of the curriculum.

### Study Design, Power Analysis and Allocation

An educational randomized controlled study was conducted in association with the curriculum as described above in September, 2011, at the Institute of Medical Biometry and Medical Informatics of the University of Freiburg, Germany. The complete curriculum included the interventional part (see below) and was completed in 5.5 days.

**Figure 2:**
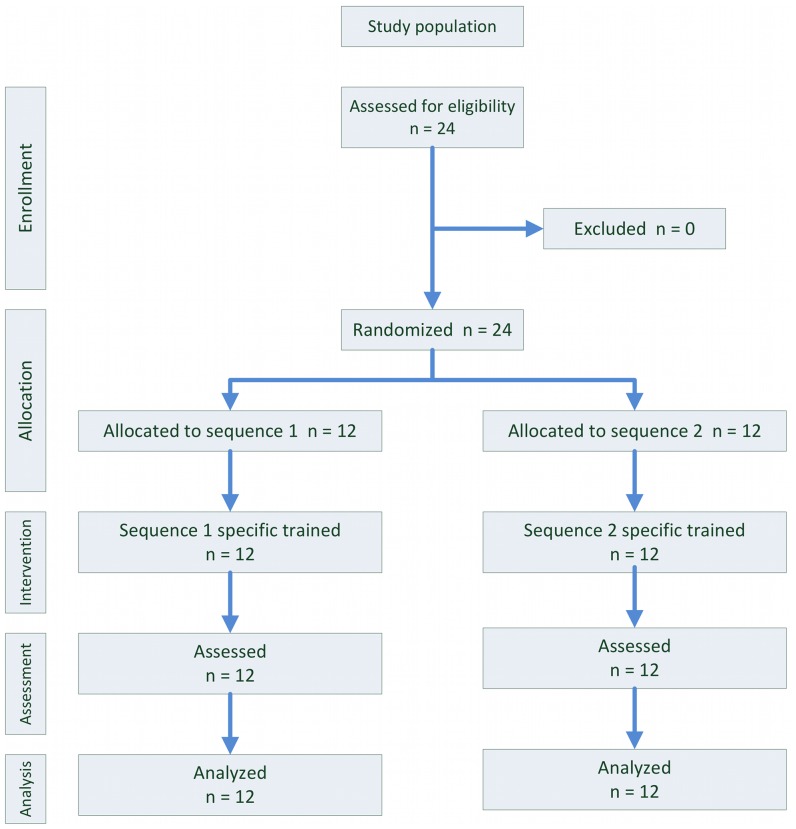
Modified CONSORT diagram. Twelve students were allocated to each group and could be analyzed.

Study design and reporting follows the CONSORT statement for randomized controlled trials in parallel group design (see Figure 2) [37].

Prior to recruitment, power analysis for trials in parallel group design was performed using the R statistical package. The estimation of standardized effect sizes (Cohens’ d = mean differences/pooled standard deviation) for similarity measures was difficult because no empirical data sets were available which allowed a calculation of the mean differences and standard deviations to be expected. A sample size of n = 12 participants per group was calculated for parallel group design under the estimation of a high effect size of d = 1.2, a power of 0.8 and a significance level of 0.05.

24 students from four European countries (Austria, Germany, Slovenia and Switzerland) were included in the study. The students were recruited from universities which offer bachelor or master degrees in biology in combination with a minor subject in computer sciences or bachelor or master degree in computer sciences in combination with a minor subject in the life sciences. In every case inclusion criteria for participation were checked: applicants combined undergraduate knowledge in the life sciences with basic knowledge in computer sciences.

Balanced randomization was performed with a pseudo random number generator from the R statistical package prior to the first interventional training sessions (see Figure 2).

### Intervention

The intervention proper was conducted after the phase of background teaching in which the introductory modules of the curriculum were taught jointly to both groups. The intervention consisted of the differential training students received for certain topics in ontology design. For the modules 10–13 and 15–16 the students were either instructed or not. The allocation of modules to each group is displayed in Table 1. Modules 10–13 are dedicated to the proper usage of top-level categories (taken from BioTop) and corresponded directly to sections in the guidelines. Group A was trained on “Process and Participation” (module 10) and on “Immaterial object” (module 12), whereas group B was trained on “Collective material entity” (module 11) and “Information object” (module 13). Modules 15 and 16 covered the application of ontology design patterns; they also corresponded directly to sections in the guideline. Here, group A received training on the Closure ODP (module 15) and group B on the Spatial disjointness ODP (module 16).

**Table 1:** Intervention and data collection of the study.

Intervention/data collection	Application of top-level ontology		Application of ODPs
**Training group A**	Module 10 (PRO) Process and participation	Module 12 (IMM) Immaterial object	Module 15 (CLO) Closure ODP
**Training group B**	Module 11 (CME) Collectivematerial entity	Module 13 (INF) Information object	Module 16 (SPA) Spatial disjointness ODP
**Test exercises both groups**	PRO: Photosynthesis, Medical diagnosing; CME: Proteinuria,Penicillin	IMM: Fetogenesis, Stomach anatomy; INF: Operation plan, Pneumonia diagnosis	SPA: Cell membranes, Stomach wall; CLO: Circulatory system, Teeth

The intervention of the study consisted of the differential training of the students in certain content areas: students in group A received training in modules 10, 12 and 15 and no training in modules 11, 13 and 16, and *vice versa* for students in group B. Training sessions were kept balanced with regard to instructor, length, difficulty and instructional format. Data were collected in the form of ontology development exercises which were distributed evenly over all content areas with two exercises per training module.

Training sessions were kept balanced with regard to instructor, length, difficulty and instructional format. The training sessions were held in parallel for both groups by two instructors for Modules 10–13. Instructors switched groups between these interventional training sessions to minimize trainer bias. Modules 15 and 16, the two training sessions on ontology design patterns, were given by the same instructor. Basically, sessions were as closely balanced as possible between the two groups for training duration, complexity and instructional structure.

The study design introduced above has a crossed interventional schema: Groups received both the same interventions (guideline-based training or no-training) but on modules with different topics, respectively. The crossed design has been chosen in this educational study to control context dependency of the effect which is introduced by the topic of the module. It is possible that an effect depends only or partly on the topic of an educational intervention and not on the intervention proper (here: training/no-training). To control this effect, more than one module (topic) was selected in each group and the design was crossed so that each group received training on different modules respectively. For practical and ethical reasons, a pure parallel group design was impossible, however theoretical possible, in which one group would have received training on all modules and the other group no training at all.

In this study, it was not intended to compare the overall effect of training between different modules. Only the effects of guideline-based training vs. no-training were compared between the groups on same modules. However, this design has some limitations discussed below.

### Outcome Measures and Instruments

*Primary outcome measure* was the mean similarity of ontology artefacts developed by the students in the assessment exercises compared with a set of gold standard artefacts provided by the authors. Prior to the experiment the authors had prepared two test exercises for each of the six interventional training topics. All exercises were given in the same format and provided with the following material: (a) an introductory text explaining the exercise; (b) an OWL file containing the necessary ontology primitives; and (c) the upper level ontology BioTopLite, a simplified version of the upper level ontology BioTop (to be imported by (b)), which corresponded to the students’ knowledge from the training sessions (individualized for each group). The learners had to arrange the ontology classes in a taxonomic order and add describing and defining axioms according to the given task. The creation of new object properties (relations) was not allowed. The contents of the tasks and their allocation to the training sessions are presented in Table 1.

In order to measure ontology similarities different instruments were combined [39]. Prior to calculation of metrics, a modified normalization approach was applied to minimize syntactic differences between ontologies, which did not affect their intended semantics [40]. Precision, recall and f-measure were determined according to Dellschaft and Staab [34], for which we have developed a software library [39]. Another library, OntoSim, was used to calculate triple-based entity similarity in combination with average linkage or minimum weight maximum graph matching (MWMGM) similarity [35], [41], [42].

*Secondary outcome measures* were the intra-group ontology similarity metrics between artefacts created by students who had either received the training or not (intra group homogeneity between ontologies). Measuring instruments were the same as for the primary outcome.

### Data Collection

Data were collected during assessment sessions held in two and a half days directly following the training sessions in the structure as presented in Table 1, in which the participants processed the exercises. After each session the individual ontology artefact produced by each student was collected as an OWL file and stored in its original state so that each assessment session yielded 24 ontology artefacts. All 12 result OWL files from each of the 24 participating students were collected, totalling 288 files.

### Statistical Analysis

A Java program was written to pre-process the files and derive metrics as described above [39]. For each measure and each exercise a tabular output file was produced so that each file contained 24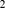 = 576 similarity/distance values corresponding to the mutual pairs of each of the 24 student results and the respective gold standard ontology.

For further aggregation, statistical procedures, and the production of graphs the R statistical package (version 2.15.1) was used [38]. The individual distance measures were aggregated by calculating means and standard deviations *for each student* inside the own group, so that aggregated measures for the distance of the ontologies in the own group resulted. Based on the individual measures for the distances to the gold standard and the calculated distances to the collection of ontologies in the own group, further parallel group analysis of aggregated means on the level of single ontologies were performed.

To estimate differences between treatment groups at the level of each ontology artefact, the treatment effect was calculated as the difference between the group means of distances to the gold standard and between the intra-group mean distances. The statistical significance was tested using the t-test for independent samples.

### Ethical Approval

Participants received an expense allowance of 500 for their participation in the training and the study. Before their agreement, students had been informed about all details of the curriculum and the following study. As part of the agreement, it was explicitly stated that the payment of the allowance was only dependent on the students’ complete attendance and full cooperation during the training sessions and the study but not on their success in the assessments or answers in the questionnaires.

Ethical approval was requested from the ethical authority of the University of Freiburg, Freiburg, Germany. The chair of the University of Freiburg ethics committee reviewed the project and concluded that a full formal ethics committee statement was not required due to the educational nature of the study. It was designed according to the general requirements for educational studies at the University Medical Center Freiburg, Freiburg, Germany, and was performed with written informed consent of the participants.

## Results

To our knowledge there are no prior studies which obtained similarity measures between ontologies developed by study participants and gold standard ontologies provided by experts after specific training on ontology design as metrics for the improvement of ontology development skills. In this randomized controlled study we sampled ontology artefacts from 24 students addressing 12 modelling task for which they had been either trained for or not prior to the assessment. We calculated similarity measures of these sample ontologies with gold standard ontology artefacts and with the artefacts of other participants inside the same training group (intra-group homogeneity).

The obtained data did not provide support for our main hypothesis, *viz.* that a guideline based ontology training improves the developers’ skills to build ontology artefacts that were more similar to pre-existing gold standards. The artefacts from the trained group were not significantly more similar to the gold standards as the ones from the untrained group. As clearly shown in Table 2, Table 4 and Table 5, we could find only very small and not statistically significant treatment effects between the trained group and the untrained group. This result is consistent for all ontology similarity metrics and ontology assessment tasks (Table 2). Even with aggregated data on topic levels only small and not significant effect sizes were observed (Table 5).

**Table 2:** Similarity with the gold standard model.

group	topic	ontology	fm	n-mtb	mtb	atb
A	CLO	tee	−0.4	−3.8	−0.1	−0.3
A	CLO	bud	6.6	0.6	−2.5	 6.9
A	IMM	sto	 11.1	1.0	 −1.5	−0.1
A	IMM	fet	−5.4	2.0	0.3	1.2
A	PRO	pho	1.6	1.6	−0.4	−2.8
A	PRO	dia	1.7	2.2	−1.0	2.4
B	CME	pru	−4.7	0.1	0.0	0.0
B	CME	pen	−3.6	−0.9	1.4	0.1
B	INF	pne	−6.0	2.0	−0.1	−1.1
B	INF	ope	1.0	1.5	−0.1	0.0
B	SPA	cem	0.5	−1.4	0.0	1.4
B	SPA	sta	7.0	4.1	−0.8	1.5

Ontology similarity metrics, displayed as absolute difference in percent between trained and untrained groups ordered by trained group, training topic and individual assessment task (for details see Table 1). The similarity/distance metrics shown are f-measure (fm), MWMGMS after normalization (n-mtb), MWMGMS without normalization (mtb) and average linkage without normalization (atb), the last three combined with triple-bases entity similarity as local measure. Significance levels of group comparisons are indicated as ∼: p
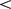
0.15, 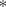: p
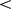
0.1, 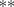: p
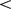
0.01, 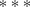: p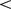.001.

**Table 3:** Effect-sizes of similarity with the gold standard ontology and intra-group similarity.

group	topic	ontology	GS-fm[%]	GS-fm d	IH-fm[%]	IH-fm d
A	PRO	pho	1.6	0.12	9.7	0.66 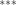
A	PRO	dia	1.7	0.12	6.5	0.35 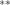
A	IMM	sto	11.1	 0.63	11.3	0.59 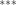
A	IMM	fet	−5.4	−0.30	2.3	0.16
A	CLO	tee	−0.4	−0.03	−3.0	−0.20
A	CLO	bud	6.6	0.51	4.1	0.31 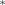
B	CME	pru	−4.7	−0.38	8.0	−0.51 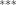
B	CME	pen	−3.6	−0.34	−5.2	−0.43 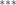
B	INF	pne	−6.0	−0.46	−2.3	−0.23 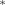
B	INF	ope	1.0	0.05	−13.0	−0.69 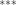
B	SPA	cem	0.5	0.04	−9.0	−0.80 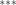
B	SPA	sta	7.0	0.37	−0.2	−0.01

F-measure ontology similarity metrics with the gold standard (GS) and f-measure intra-group homogeneity (IH), displayed as absolute differences in percent between trained and untrained groups and Cohens’ d effect sizes. Ordering is by trained group, training topic and individual assessment task. For details on abbreviations and symbols see Table 2.

**Table 4:** Aggregation of effect-sizes on topic level.

group	topic	GS-fm [%]	GS-fm d	IH-fm [%]	IH-fm d
A	PRO	1.6	0.12	8.1	0.49 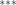
A	IMM	2.8	0.16	6.8	0.40 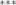
A	CLO	3.1	0.23	0.5	0.04
B	CME	−4.1	−0.36	−6.6	−0.47 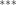
B	INF	−2.5	−0.16	−7.7	−0.50 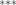
B	SPA	3.7	0.24	−4.6	−0.23 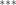

F-measure ontology similarity metrics with the gold standard (GS) and f-measure intra-group homogeneity (IH) aggregated on topic level, displayed as absolute differences in percent between trained and untrained groups and Cohens’ d effect sizes. Ordering is by trained group and training topic. For details on abbreviations and symbols see Table 2.

**Table 5:** Intra-group similarity (homogeneity).

group	topic	ontology	fm	n-mtb	mtb	atb
A	PRO	pho	9.7 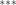	0.1	−0.5 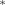	−0.3
A	PRO	dia	6.5 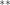	0.1	−0.8 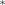	−0.9
A	IMM	sto	11.3 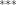	−0.3	0.0	 −1.3
A	IMM	fet	2.3	−1.2	0.0	0.4
A	CLO	tee	 −3.0	−0.7	0.0	−0.4 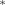
A	CLO	bud	4.1 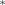	0.4	1.0	−1.0
B	CME	pru	−8.0 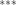	0.1 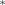	0.0	0.0
B	CME	pen	−5.2 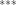	−1.4 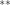	0.3	 1.0
B	INF	pne	−2.3 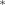	−0.2	0.0	0.1
B	INF	ope	−13.0 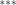	−0.7	0.1	 0.3
B	SPA	cem	−9.0 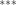	0.3	0.0	−0.2
B	SPA	sta	−0.2	1.1	−0.7	−0.2

Intra-group ontology similarity metrics (homogeneity), displayed as absolute difference in percent between trained and untrained groups ordered by trained group, training topic and individual assessment task. For details on abbreviations and symbols see Table 2.

Our second hypothesis that the homogeneity between ontologies from different developers would increase due to guideline based training when compared with the untrained group was not supported by our data either (Table 3, Table 4 and Table 5). Although we observed moderately significant effect sizes for the f-measure intra-group similarity, the overall comparison between the groups is not conclusive. For group A the f-measure increases significantly about 5% (absolute) after training but for group B it decreases significantly about 6% (Table 5). For the other tested similarity measures only small differences between the trained and untrained group of lower than 1.5% were found. Why the f-measure metrics are more sensitive for intra-group similarity differences than the other metrics remains unclear and should be subject of further investigation. Contrary to our prior assumptions, we did observe differences in intra-group similarity between trained and untrained students *in both directions*. However, the direction of the differences might depend on the topic of the training session and corresponding assessment tasks or the (random) allocation of students to the groups. The exact cause is however not determinable with our study design.

At this point, we are not able to confirm our assumption that skills of novice ontology developers are improved by guideline-based training. Moreover, our results indicate that the content topic of the training and the corresponding assessment task are factors that might dominate the training effects. Furthermore, despite our best efforts those content topics may have been distributed in an imbalanced way in the two groups and may have caused the different effects in the intra-group f-measure similarity metrics.

## Discussion

Our research question for this randomized controlled trial was whether the skills of ontology developers can be improved by a guideline-based training when compared with developers who received unspecific training. To provide empirical evidence to support this hypothesis, we have developed a curriculum based on a guideline on good ontology design and evaluated its effect in a randomized controlled trial. The *primary outcome parameter* of the study was the similarity of ontologies developed by participants with a gold standard model provided by the authors of the guideline. The *secondary outcome parameter* was the intra-group homogeneity of ontologies.

The data of our study did not provide evidence for either hypothesis; neither did the similarity to a gold standard model increase significantly nor did the intra-group homogeneity of participants change consistently.

There are several possible causes for the difficulty to detect an unambiguous effect of our training on the quality of ontologies as the product of the development process. On the one hand, the explanatory power of the study was limited due to the study design. However, this argument does not account for the failure to even detect a difference in similarity to the gold standard model. On the other hand the deployed similarity metrics might be too insensitive to detect slight differences in features present in our ontologies.

The simplest explanation for the result of our study would be that students were overloaded with too much information in too short a time. The complete interventional training sessions were given in 2.5 days. Although they consisted of two 105 min units for each instructional topic and an additional free training session, they were overly ‘packed’ with information. Thus, students possibly had not enough time to consolidate their recently acquired knowledge in a longer training session. Moreover, the recall of fresh knowledge and skills might even be inhibited by the task to tackle new problems after only a short interval to a prior learning experience that was not yet fully consolidated (retroactive interference) [43].

However, the results of our study should not be considered to indicate a complete failure of the guideline-based approach of ontology development. As an evaluation of a guideline, our study had objectives on two levels. First, it had the objective to provide evidence on the efficacy of a guideline-based training on the performance of ontology developers. On top of that, a secondary objective was to show that the guideline-based approach is superior to the ‘conventional’ more or less unguided development of ontologies. What we can state here, is that our training was not effective, but that does not imply that the guideline-based approach, in general, is not effective. As we outlined above, our interpretation is that the educational intervention and the measurement instrument were insufficient to show small differences in a complex environment.

### Limitations of the Study

As an educational study in a complex environment, this study has some limitations which render the interpretation of its results very difficult.

From a pure educational standpoint, a comparison of an educational intervention with an unspecific instruction in the control group was criticized to be low-stakes educational research [44]. Arguing from this standpoint alone, a positive result would have meant only that the provided educational intervention ‘had been better than nothing’ – which is really not much. However, as a study in guideline evaluation we designed it to show the effectiveness of the guideline-based approach. Seen from this more complex standpoint, a positive result could have been interpreted as: (1) the provided guideline-based intervention ‘had been better than nothing’, and (2) the guideline-based approach to ontology engineering is better than a non-guided approach.

We faced the problem to show a clear effect under the condition of a small sample size and a complex educational and technical setting. However, a larger sample size was not feasible due to practical limitations. It was already difficult to recruit the participants of the present study, and a larger group size would also have impeded the educational implementation. There would have been two alternative study designs with higher statistical power:

1. Within the parallel group design we could have chosen to assign all interventions to the same group. Most probably this would have decreased the spread of our results and thus increased the statistical power of the result. However this would have had two important disadvantages: It would have been a hindrance for recruitment, as half of the participants would have learned considerably less during the summer school (and learning is one of the key incentives for participation in such an event). Moreover, this line would have increased the ‘better than nothing’ problem of educational studies.
2. Statistical significance would have also been higher, if we had evaluated our data by using the crossover method [45], [46]. Although the conditions were kept as closely similar as possible for the two groups, a major structural limitation of an educational crossover design lies in the training and assessment of different content areas. When sequential crossover studies are performed in pharmaceutical studies, a washout period can be inserted that is long enough to reach complete extinction of the treatment effect of the first treatment period. In educational crossover studies, however, a back-learning (forgetting) is neither possible nor desirable, so that the instructional methods that are to be compared must be applied to different content areas. Although we switched the roles of intervention group and control group for different treatments, we decided not to analyse the results as a crossover study because we considered the topical differences of the educational interventions as too large.

Even this way, and however carefully chosen for similarity in difficulty, length and amount, the content itself introduces a confounding variable in our design in which training for different content was ‘switched’ between groups. Although we tried to match the training topics of ontology design to have comparable content distributions for each group, they remain markedly different due to the character of the topics (Table 1). We consider this heterogeneity in content a possible reason for the different results on intra-group homogeneity in the two groups. As further elaborated in the next section, no validated and tested evaluation methods for computerized measurement of quality indicators of a larger number of ontologies are available. The resources of this project limited us to employ existing methods without prior prototyping of the study. Thus we missed the possibility to detect the limitations of the ontology similarity/distance metrics which were originally introduced for the evaluation of ontology mapping experiments.

### Evaluation of and Metrics for Formal Ontology

Due to the complex nature of the ontology artefacts, their evaluation is inherently difficult and manifold. Furthermore, there are often multiple correct ways to represent the same facts, and the objectives and methods of ontology evaluation are numerous [32], [47], [48]. What do we exactly mean when we talk about ‘good ontology’? This property might be dependent on the objectives of the ontology under scrutiny, its philosophical foundations and the intention of the investigator. It is possible for two serious investigators to rate the same ontology on opposite poles of a quality scale [49].

In our view, the quality criteria for ontologies should be defined in accordance with their real or hypothetic application scenarios, besides the mere formal correctness of an ontology, which is already a starting point for good quality. However, as our interest lies in biomedical ontology, we can define several parameters for good quality ontology which are independent of their primary intended application scenario:

- Consideration of a continuous and appropriate ontological commitment throughout the ontology development process. The integration of a top-level ontology [21], [50]–[52] in the domain ontology can guarantee for this adherence in a more robust way as the usage of certain ontology design patterns, as the top-level ontology already provides many patterns in an embedded way, ensuring all patterns harmonize with each other, i.e. fit a common scope and development philosophy.
- Adherence to coding standards and naming conventions as defined by policy and best practice providers [8], [53].
- Correct and exhaustive representation of the domain that is intended to be represented.

Although a variety of metrics and methods could be useful in ontology evaluation, it is not clear how to *measure* certain properties, which might be used as quality indicators as listed above. In the case of our experiment, we can judge the quality of the produced ontologies by observing selected features such as the structure of the taxonomy, the axiomatization, representational errors, completeness, and correctness. However, to quantitatively assess the structure of the taxonomy or axiomatization in an objective and automatically manner seems to be impossible with the given tools. Our measurement hypothesis to compare the learners’ ontology artefacts with a gold standard, and to apply ontology similarity metrics, seemed most adequate to capture the two latter points. Unfortunately, we cannot claim that this strategy was successful. In the end, these methods, which were originally developed for ontology alignment experiments, were too insensitive to detect relevant differences between the test and gold standard ontologies.

### Further Research

Future research should develop ontology evaluation methods that are able to quantify quality parameters of ontologies in accordance with a specific description logic expressivity regime (affected by the application use case and reasoning style). These quality parameters must be empirically validated for their explicit scopes. One direction of ontology evaluation can be the deployment of competency questions [54] which is a way to formalize requirements and assess their fulfilment. An automatic and objective evaluation of ontology may be possible with a strong formalization of competency questions as logical expressions.

Further research should also yield empirical evidence for the efficacy of certain beliefs, dogmas and practices in ontology development. Ontology development is not an ‘artistic craft’ but an engineering activity which should be led by a high level of evidence and not only by expert opinion in analogy to practice in evidence based medicine [55].

How should *quality indicators* for ontologies be operationally defined? The overall quality of an ontology is dependent on a variety of factors. Hence, quality indicators should at least be defined to provide useful measures for ontology development and deployment; furthermore they should be unambiguous and *measurable* in a reproducible way, ideally automatically to avoid any bias. How can the quality of ontologies be measured computationally? Although many methods for ontology evaluation have been described or proposed, many open questions remain on how to measure quality indicators of ontologies technically, reproducibly and automatically. To support good quality ontology design, there should be an easy-to-use toolbox of ontology metrics which can be applied from the ontology editor of choice. Using such a toolbox, the developers can evaluate their ontology artefacts at each step of the development process and judge on how to proceed.

How effective are specific *ontology designs*? It is not enough to claim that a certain type of development, top-level ontology, taxonomy or ontological commitment will result in better ontologies. At present, such claims are often brought forward without any empirical evidence. To overcome this situation, it is desirable to provide empirical evidence which methods of ontology design are more effective to reach specific objectives.

How can the *behaviour of ontology developers* be changed effectively? Evidence on good practice is not enough. Ultimately, good practices and new methods of development have to be implemented in the ‘community of developers’. To understand and guide those complex socio-technical processes it must be understood how to change thinking, behaviour, communication and tools of developers. To investigate this complex network more educational studies are necessary. Furthermore, this educational research must adhere to the premise of proceeding incrementally by strictly changing only one parameter at a time in the experimental setting of each study [56].

## Conclusions

Inadequate knowledge and insufficient skills of ontology developers are among the causes for quality problems of ontologies in the biomedical domain. To improve this situation, we developed GoodOD, a guideline for good ontology design, optimized for the development of OWL ontologies using description logics for representation and reasoning. We implemented a training course based on this guideline. In a randomized controlled trial with 24 students we investigated the efficacy of the guideline based training on the quality of developed ontology modules and patterns.

We could not detect enhanced quality in terms of similarity to gold standard representations of the ontology artefacts produced after the selective training, when compared to artefacts built without prior training. Due to the study design outlined in this paper, the interpretation of such study results is difficult. Although we could not provide evidence for the effectiveness of a guideline-based approach to ontology design, the study neither provides evidence against the efficiency of this approach. More effort has to be invested in further research which can reliably provide empirical evidence whether specific development guidelines and practices in ontology design are effective.
